# An Exploratory Investigation of Goal Management Training in Adults With ADHD: Improvements in Inhibition and Everyday Functioning

**DOI:** 10.3389/fpsyg.2021.659480

**Published:** 2021-09-09

**Authors:** Daniel André Jensen, Anne Halmøy, Jan Stubberud, Jan Haavik, Astri Johansen Lundervold, Lin Sørensen

**Affiliations:** ^1^Department of Biological and Medical Psychology, University of Bergen, Bergen, Norway; ^2^Division of Mental Health, Betanien Hospital, Bergen, Norway; ^3^Division of Psychiatry, Haukeland University Hospital, Bergen, Norway; ^4^Department of Clinical Medicine, University of Bergen, Bergen, Norway; ^5^Department of Psychology, University of Oslo, Oslo, Norway; ^6^Department of Research, Lovisenberg Diaconal Hospital, Oslo, Norway; ^7^Department of Biomedicine, University of Bergen, Bergen, Norway

**Keywords:** ADHD, goal management training, treatment, intervention, inhibition, non-pharmacological, executive functioning

## Abstract

**Background:** Adults with attention deficit/hyperactivity disorder (ADHD) are predominantly treated with medication. However, there is also a need for effective, psychologically based interventions. As ADHD is strongly associated with reduced inhibitory control, cognitive remediation approaches should be efficient. Goal management training (GMT) aims at enhancing inhibitory control and has shown positive effects on inhibitory control in non-ADHD patient groups. The aim of the current study was to explore whether GMT would specifically enhance inhibitory control in adults with ADHD, and if such an enhancement would lead to secondary improvements in self-reported everyday functioning.

**Methods:** Twenty-one participants with ADHD (mean age: 39.05 years [SD 11.93]) completed the intervention and assessments pre-, post- and 6 months after the intervention. Measures included neuropsychological tests and self-report questionnaires pertaining to cognitive- and executive functioning, emotion regulation, quality of life, and ADHD symptoms.

**Results:** Compared to baseline, the participants showed enhanced inhibitory control on performance-based measures at post-assessment and 6-month follow-up. The participants also reported increased productivity and reduced cognitive difficulties in everyday life at both assessments post-treatment, as well as improvements in aspects of emotion regulation and a reduction in the severity of core ADHD-symptoms at 6-month follow-up.

**Conclusion:** Our exploratory study showed that GMT seems to specifically improve one of the core executive dysfunctions in ADHD, namely inhibitory control, with a positive effect lasting at least 6 months post-treatment. The adults with ADHD also reported improved self-regulation in their everyday life after completing GMT, providing strong arguments for further investigations of GMT as a treatment option for this group of adults.

**Clinical Trial Registration:** The study is registered under ISRCTN.com (ISRCTN91988877; https://doi.org/10.1186/ISRCTN91988877).

## Introduction

Attention deficit/hyperactivity disorder (ADHD) is a common neurodevelopmental disorder affecting individuals of all ages with an estimated prevalence of 2.5–4.4% among adults (DSM-5; Kessler et al., 2006; Simon et al., 2009; Badre, 2011; American Psychiatric Association, 2013; Polanczyk et al., 2014). Many negative consequences of ADHD have been documented ranging from difficulties regulating automatic and controlled cognitive processes, including reading, in childhood (Capri et al., 2020; Mohammadhasani et al., 2020) to underachievement in work and education later in life (Biederman et al., 2006; Halmøy et al., 2009; Klein et al., 2012; Halleland et al., 2015), difficulties related to social functioning (Biederman et al., 2006; Klein et al., 2012), and even increased mortality (e.g., Dalsgaard et al., 2015). These difficulties are partly related to the core ADHD symptoms of inattention, hyperactivity and impulsivity (Badre, 2011; American Psychiatric Association, 2013). However, they have also been linked to another frequently observed characteristic of ADHD, namely reduced executive functioning (e.g., reduced academic achievement, reduced occupational attainment; Barry et al., 2002; Doyle, 2006; Raggi and Chronis, 2006; Sarkis, 2014; Halleland et al., 2015; Fabio and Caprì, 2017).

The main treatment for adults with the disorder is pharmacotherapy with stimulant drugs (Kooij et al., 2010; Faraone et al., 2015; NICE, 2018). Despite strong evidence for its effectiveness (e.g., Cortese et al., 2018), pharmacological treatment of ADHD does not seem to fit all. In fact, a substantial number of patients continue to struggle with their symptoms, are ineligible or do not tolerate the side effects of the medication, opt out of such treatment, or discontinue the prescribed treatment (Lopez et al., 2018; NICE, 2018; Mohr-Jensen et al., 2020). Among the most frequent side effects of such treatments are loss of appetite, headaches, abdominal pain, increased heart rate and blood pressure, and available findings indicate that a significant number of those receiving medication discontinue treatment due to such adverse effects (Cortese et al., 2018; Storebø et al., 2018; Elliott et al., 2020). Other investigations also indicate that medication use is associated with psychological adverse effects such as an experience of altered cognition, reduced creativity, increases in emotional difficulties, reduced engagement in activities and a sense of changing as a person (Kovshoff et al., 2016). There is also a lack of knowledge regarding long term tolerability and effects of ADHD-medication, and the risk of less common side effects (Elliott et al., 2020). Development of other treatment alternatives is, therefore, warranted. This is also in line with the stated wishes of adults with ADHD, and with findings showing that patients who are offered treatment options in addition to pharmacotherapy are more satisfied with the health services they receive compared to adults without such options (Solberg et al., 2015). Thus, there has been an increasing effort to develop psychologically based treatment alternatives for adults with ADHD (Kooij et al., 2010; Franke et al., 2018; Lopez et al., 2018; López-Pinar et al., 2018; Lam et al., 2019; Nimmo-Smith et al., 2020), most of which are based on cognitive-behavioral approaches. Findings indicate that such interventions may lead to reductions in core symptoms, and further, that cognitive remediation interventions may specifically improve the ability to organize everyday activities (e.g., Stevenson et al., 2002; De Crescenzo et al., 2017; Nimmo-Smith et al., 2020). The evidence in favor of these interventions is, however, still scarce.

This has led to efforts aimed at exploring whether psychological interventions can ameliorate difficulties in executive functioning in individuals with ADHD. Procedures to improve working memory functioning have been investigated in children (e.g., Melby-Lervåg and Hulme, 2013) and adults with ADHD (e.g., Dentz et al., 2020). Although some studies show positive short-term effects on working memory functioning, long-term effects are uncertain and there is limited support for generalization to other aspects of executive functioning (Melby-Lervåg and Hulme, 2013; Dentz et al., 2020). The viability of applying existing working memory training programs on a population of adults with ADHD has also been questioned due to indications of limited tolerability (Marcelle et al., 2018). Similarly, studies of neurofeedback as a treatment for ADHD in children have yielded mixed findings with regards to effects on core symptoms and executive functioning (Cortese et al., 2016; Van Doren et al., 2019), while studies of neurofeedback in adults with ADHD are still limited.

In an effort to further the understanding of whether psychological interventions targeting executive functioning in adults with ADHD would be an efficient treatment alternative, we wanted to examine the effects of goal management training (GMT; Robertson, 1996; Levine et al., 2000, 2011). GMT is a group-based, metacognitive remediation protocol with an emphasis on strengthening inhibitory- and attentional control to support participants in employing strategies to maintain goal-directed behavior over time. Thus, the choice of GMT in the current study was based on the fact that poor inhibitory control has been described as a predominant causal factor of ADHD (e.g., Barkley, 1997; Sonuga-Barke et al., 2010), in addition to being conceptualized as a core component of executive functioning (Badre, 2011; Miyake and Friedman, 2012). It is also a strength that GMT has been shown to ameliorate executive dysfunction, including inhibitory control, in other groups (e.g., older adults, patients with substance use disorders) experiencing some of the same challenges as adults with ADHD (van Hooren et al., 2007; Alfonso et al., 2011; Stamenova and Levine, 2019). To the best of our knowledge, GMT for adults with ADHD has only been tested in a small-scale pilot study (In de Braek et al., 2017). In that study, a modified manual consisting of GMT and psychoeducation (*n* = 12) was compared with the effect of psychoeducation without GMT (*n* = 15), and it included outcome measures focusing predominantly on everyday cognitive functioning according to the Cognitive Failures Questionnaire (CFQ; Broadbent et al., 1982) and a clinician-rated evaluation of everyday cognitive functioning (see Schneider et al., 1997). Only one performance-based measure, assessing everyday problem-solving, was included (Zoo Map From the Behavioral Assessment of the Dysexecutive Syndrome; Krabbendam et al., 1999). A positive effect of GMT was found only on the clinician-rated evaluation of cognitive functioning in everyday life (In de Braek et al., 2017). Importantly, the study employed a modified version of GMT which included several sessions of psychoeducation and an individual session in addition to the sessions that are part of the GMT manual. As such, it is difficult to draw conclusions about the specificity of the reported effects to GMT as psychoeducation may also have made significant contributions (e.g., Vidal et al., 2013).

The aim of the current study was to follow up on the pilot study by In de Braek et al. (2017) by employing an unmodified version of GMT. We aimed to test a neuropsychological model of how to measure effects of GMT in adults with ADHD by including performance-based measures of attention and executive functions, and by this provide a model that can guide future randomized controlled studies of GMT. Previous studies in other patient groups have found significant improvements following GMT on several performance-based measures of inhibitory control and related constructs (e.g., Levine et al., 2011; Stubberud et al., 2013; Hagen et al., 2020), including on a self-report measure of inhibitory control and executive functioning (Stubberud et al., 2014). We therefore wanted to test whether GMT specifically targets the typically found reduced inhibitory control characteristic of adults with ADHD. To do so, we conducted an exploratory pilot trial of GMT in adults with ADHD, focusing specifically on effects on inhibitory control compared to effects on other aspects of executive functions such as working memory, flexible control of processing speed, and general problem-solving. We expected to find significant effects of GMT predominantly on test measures assessing inhibitory control, such as the Color Word Interference Test (CWIT) and the Tower test (Delis et al., 2001). These are tests that have previously shown that adults with ADHD tend to have impaired inhibitory control (see Young et al., 2007; Halleland et al., 2015). As secondary aims, we wanted to examine aspects of everyday functioning and expected that improved inhibitory control following GMT would be reflected in self-reports of executive-, behavioral-, and emotional control, as well as in improved quality of life.

## Materials and Methods

### Participants

In total, 36 potential participants were recruited for the present study through two different approaches. A small subset of participants (*N* = 7) were recruited through an existing study of ADHD in adults at the University of Bergen (see Halleland et al., 2012 for a description of this study), while the majority of participants (*N* = 29) were recruited through local outpatient clinics in the municipality of Bergen. Recruitment was conducted by distributing a short information letter about the project, i.e., inclusion and exclusion criteria, the GMT intervention, assessments as well as a prompt to contact members of the project staff for further information. Upon contacting a project member, interested individuals were screened for eligibility and given further information about the study as well as an informed-consent form in accordance with the Helsinki declaration. The study protocol was approved by the Regional Committee for Medical and Health Research Ethics, West Norway (2015/2325). All participants were compensated with 1000 NOK (approximately 110 USD) at the completion of the follow-up assessment to cover travel expenses.

Inclusion criteria for the study were an age of 18 years or older and a clinical diagnosis of ADHD (obtained prior to the project). Participants on medication were asked to avoid changes in dosage during the project period unless necessary. Exclusion criteria for the project were a lifetime history of psychotic disorder or an ongoing, severe psychiatric illness (i.e., moderate to severe suicidality, severe depression, severe social anxiety preventing participation in group sessions), ongoing substance use disorders and a full-scale intelligence quotient (IQ) below 80.

### Procedure

All potential participants completed the Mini International Neuropsychiatric Interview Plus (M.I.N.I. Plus; Sheehan et al., 1998) as the first step of the baseline assessment to screen for severe psychiatric disorders or substance abuse. The Wechsler Abbreviated Scale of Intelligence (WASI; Wechsler, 1999) was used to estimate the participants’ IQ. After completion of this introductory step, eligible participants were asked to complete the assessment procedure (see details below). With one exception (i.e., one participant had to be moved to a different group due to scheduling conflicts and therefore completed the assessment 5 weeks before attending the first session of GMT), all assessments were conducted within 3 weeks prior to the first session of GMT. Post-treatment assessments were conducted within two weeks following the last group session, and the follow-up assessment was conducted six calendar months after completion of the intervention (±2 weeks).

### Goal Management Training

Goal management training is a group-based metacognitive remediation program developed by Robertson (1996), Levine et al. (2000) based on Duncan (1986). According to Duncan’s theory goal management fails as a result of the individual being unable to maintain current goals when faced with competing demands in the form of external or internal stimuli. GMT therefore emphasizes a five-stage strategy aimed at supporting the processes needed for goal achievement. These stages include the intermittent stopping of ongoing behavior to assess whether this is in line with current goals and, indeed, whether current goals are clear, structuring goals as a manageable set of subgoals, self-cueing to regulate alertness and attentional control, and regular reassessment of goal list and the progress made as a result of current behavior. Mindfulness-based exercises are also included to support sustained attention, help participants maintain a present centered focus and self-regulation. The intervention followed a manualized protocol used in earlier studies (e.g., Stubberud et al., 2013; Tornås et al., 2016) consisting of PowerPoint slides and a participant workbook. The materials used in this study were translated into Norwegian and back-translated to English as part of the study conducted by Stubberud et al. (2013). Minimal adjustments were made to the materials in order to adapt the educational part to participants with ADHD (i.e., mention of brain injury and its consequences were replaced with references to ADHD). The intervention consisted of nine weekly 2-h group sessions (see Table 1). Of note, participants had to attend a minimum of six out of the nine group sessions to be classified as completers. Each group had four to eight participants and was led by a clinical psychologist and a co-therapist who was either a clinical psychologist or a clinical psychology student with clinical experience. The sessions were conducted during nine consecutive weeks when possible, or over a maximum of 11 weeks when holidays made this necessary. The sessions consisted of lectures, discussions and skill training intended to increase participants’ awareness of their own attention as well as their awareness of the skills and techniques included in GMT. The included strategies are aimed at promoting goal-directed behavior through increasing executive and inhibitory control, stressing participants to periodically stop ongoing behavior (“stop-and-think”), monitor performance, and employ a stepwise approach to problem-solving (Levine et al., 2011). Furthermore, the element of sustained attention runs continuously through GMT, and is reinforced through mindfulness exercises (Kabat-Zinn, 1990). Participants were also encouraged to practice between sessions and to employ the workbook to structure these efforts. Homework assignments included monitoring everyday behavior, recording absentmindedness as well as goal attainment, and mindfulness exercises. These assignments were the basis for in-group discussions of the participants’ experiences related to executive difficulties in their everyday life.

**TABLE 1:** An overview of the nine GMT sessions as well as the main content.

GMT session	Description and content
Session 1: The Present and the Absent Mind	Introduction of the concepts of present- and absentmindedness, as well as relating absentmindedness to failure of goal-attainment in everyday life. Introduction of Mindfulness (“body scan”) as a tool to promote present-mindedness. Participants are asked to monitor absentmindedness and to practice mindfulness between sessions
Session 2: Absentminded Slip-Ups	Factors which promote or reduce the likelihood of absentminded slip-ups and consequences of such slip-ups are discussed. Participants are asked to continue their monitoring. Mindfulness exercises extended by introduction of a breathing exercise which they are asked to practice between sessions for the remainder of the intervention
Session 3: The Automatic Pilot	“The automatic pilot” is introduced as a descriptor of absentmindedness characterized by following existing routines. Discussion of how this may lead to unwanted responses. Participants are asked to log situational factors which increase the chances of slip-ups between sessions
Session 4: Stop the Automatic Pilot	“STOPPING!” the automatic pilot is introduced as a strategy for increasing present-mindedness and monitoring current behavior and mental content. “STOPPING!” is practiced between sessions
Session 5: The Mental Blackboard	Checking is introduced as a metaphor for working memory and as another key concept for increasing goal-attainment. The notion of limited capacity and the risk of having important information overwritten is emphasized. Checking the content of working memory is introduced along with a shortened breathing exercise in the “STOP!-FOCUS-CHECK” technique
Session 6: State Your Goal	Explicitly STATING relevant goals and behaviors is introduced as a strategy to promote retention of goals in working memory. “STOP!-STATE” cycle practiced
Session 7: Making Decisions	The concept of goal-conflict is introduced and discussed, as well as practical and emotional consequences. A To-Do list is introduced as an aid both for retention of goals and to alleviate decision-making. Use is incorporated in the “STOP!-STATE” cycle
Session 8: Splitting Tasks into Subtasks	Modification of overwhelming tasks by dividing these into manageable subtasks is discussed and practiced using the “STOP!-STATE-SPLIT” technique. Participants are asked to continue practice between sessions
Session 9: Checking (STOP!)	Checking, or the concept of adapting current goals and ongoing behavior as a result of changes in the external or internal environment, is discussed and practiced. Content and experiences from the program are summarized

### Clinician Administered Measures

The M.I.N.I. Plus (Sheehan et al., 1998) was used to assess potential participants for severe psychiatric illness necessitating exclusion from the project. The M.I.N.I. Plus was administered at baseline by project members who were either a licensed clinical psychologist or a clinical psychology student with experience from clinical practice and the use of diagnostic interviews under the supervision of a clinical psychologist.

### Performance-Based Measures

#### Wechsler Abbreviated Scale of Intelligence

Performance on two subtests from WASI (Matrix reasoning and Vocabulary: Wechsler, 1999) were used to estimate the participants’ IQ score. Participants completed this measure at baseline.

#### Delis-Kaplan Executive Function System: Trail Making Test

The Trail Making Test (TMT) from the Delis-Kaplan Executive Function System (D-KEFS; Delis et al., 2001) was administered to assess the executive functions of attentional control and cognitive flexibility (switching). The fourth task is of special interest in the current study. Here, the participants are asked to connect circles with numbers and letters in an ascending and alternating pattern. Completion time and number and type of errors are recorded.

#### Delis-Kaplan Executive Function System: Color-Word Interference Test

The CWIT (Delis et al., 2001) was administered to assess the executive function of inhibitory control. The test consists of 4 different subtasks. Of interest to the current investigation are conditions three and four where participants are instructed to name the color of a color-word printed in a color that does not match the color-word, or to switch between naming the un-matched, printed color and reading the color words. Completion times and errors are recorded.

#### Delis-Kaplan Executive Function System: Tower Test

The Tower Test (Delis et al., 2001) was administered to assess inhibition. In the test, subjects are asked to recreate a model based on a picture of the required outcome. To do so they are asked to use a specified number of disks of varying sizes and place them in the depicted pattern. They may only move one disk at a time, all disks must always be placed on one of three pegs, and larger disks may not be placed on top of smaller disks. Participants are asked to complete the depicted model in as few moves as possible, while attending to the rules. Time of first move, number of moves, rule violations, completion time and performance (i.e., completion of correct model) are recorded.

#### Letter-Number Sequencing and Spatial Span

The Letter-Number Sequencing task from the Wechsler Adult Intelligence Scale – 4th edition (Wechsler, 2008a) and the Spatial span from the Wechsler Memory Scale – 3rd edition (Wechsler, 2008b) were used to assess the participants’ working memory functions. In the Letter-Number Sequencing an increasing number of letters and numbers are read to the participant, the participant is then asked to repeat the sequence by arranging the numbers in increasing order followed by the letters in alphabetical order. In the Spatial span task, the examiner touches a sequence of blocks in a specified order and the participant is asked to copy the sequence (i.e., forward span) or to do so in the opposite order (i.e., backward span).

#### Hotel Task

The Hotel task (Manly et al., 2002) was administered as an analog of real-life problem-solving and a measure of generalization. The Hotel task consists of six different subtasks, and the participant are asked to distribute the allotted time of 15 min as evenly as possible across five of these while also completing the sixth task at two specified time points. Deviations from ideal time (e.g., 300 s) spent on the five time-demanding subtasks are recorded, so is deviation from the specified time when completing the sixth task, as well as total number of tasks attempted. The Hotel task has been shown to be sensitive to executive dysfunction and to have acceptable ecological validity (Roca et al., 2009).

### Self-Report measures

#### Cognitive Failures Questionnaire

The CFQ (Broadbent et al., 1982) is a 25-item self-report questionnaire where respondent are asked to rate each statement using a scale from 0 (never) to 4 (very often). Higher total score indicates a higher number of difficulties related to failures in perception, memory, and motor functions.

#### Adult ADHD Self-Report Scale

The Adult ADHD Self-report Scale (ASRS; Kessler et al., 2005) is an 18-item symptom checklist assessing the presence of core symptoms of ADHD during the last 6 months prior to evaluation. Respondents are asked to scale each item from 0 (never) to 4 (very often). The checklist consists of nine statements related to symptoms of inattention and nine statements related to symptoms of hyperactivity/impulsivity. Both sub-scores for these two domains as well as a total sum score are calculated.

#### Wender-Utah Rating Scale

The Wender-Utah Rating Scale (WURS; Ward et al., 1993) is a 25-item retrospective self-report checklist assessing the presence of various difficulties associated with ADHD in childhood based on the Utah criteria (Wender, 1972). Respondents are asked to respond to each item using a scale ranging from 0 (not at all, or just a little) to 4 (very much). The WURS was used to characterize the sample and was only administered at baseline.

#### Adult ADHD Quality of Life Inventory

The Adult ADHD Quality of Life inventory (AAQoL; Brod et al., 2005) is a 29-item questionnaire were participants were instructed to respond to each item using a 5-point scale ranging from 1 (not at all/never) to 5 (extremely/very often), resulting in four subscales (Life Outlook, Life Productivity, Psychological Health, and Relationships) as well as a total score.

#### Behavior Rating Inventory of Executive Function – Adult Version

The Behavior Rating Inventory of Executive Function Adult version (BRIEF-A; Gioia et al., 2000) is a 75-item self-report measure of everyday executive function. Participants are asked to rate each item ìs frequency of occurrence on a 3-point Likert scale from 1 (never) to 3 (often). The instrument yields nine clinical scales, as well as two broad index scores. Of particular interest to the current study are the subscales Inhibit, Shift and Working memory, as well as the index scores for Behavior regulation and Metacognition. The Global executive composite score is also reported.

#### Dysregulation of Emotions Rating Scale

The Dysregulation of Emotions Rating Scale (DERS; Gratz and Roemer, 2004) is a 36-item questionnaire where participants are asked to rate each item using a 1 (almost never) to 5 (almost always) scale. The DERS consists of six subscales as well as a total score. For the present study, subscales measuring difficulties engaging in goal-directed behavior, impulse control difficulties and access to emotion regulation strategies when experiencing challenging emotions as well as total score, were employed.

All tests and questionnaires, except the WASI and the WURS, were administered at all assessments. Cronbach’s αs ranged from acceptable to excellent (between 0.87 and 0.96).

### Analyses

#### Preliminary Analyses

Statistical analyses were conducted using R version 4.0.2 (R Development Core Team, 2020) and SPSS version 25 (Ibm Corporation., 2017). For all variables containing missing items, comparisons of means and covariances were conducted using Little’s missing completely at random test (Little, 1988). Outliers were identified using median absolute deviation (MAD) and a conservative cut-off of ± three times the MAD (Leys et al., 2013). Independent sample, two-sided *t*-tests were conducted as preliminary analyses to compare the baseline characteristics of those participants who completed the intervention and those who dropped out with regards to self-report- and performance-based measures.

#### Main Analyses of Treatment Effects

Linear mixed-effects regression was performed using the lme4 package for R (Bates et al., 2015). Random intercepts were specified, and restricted maximum likelihood (REML) was used. Assessment session was used as the metric of time in the analyses, and coefficients represent change from baseline. Due to the limited statistical power of the study, these analyses were conducted without controlling for covariates. In a second step, exploratory analyses including medication status and age as covariates were conducted on those measures showing a significant effect in the principal analyses. Significance tests were adjusted using false discovery rate control due to the number of tests performed following the procedure of Benjamini and Hochberg (i.e., *p* < *d* × *i*/*n*; Benjamini and Hochberg, 1995; Glickman et al., 2014). Power-analyses conducted using G^∗^power (Faul et al., 2007) prior to the study indicated that with an assumption of medium effect sizes and an α of.05 a total sample size of 27 participants would be required to reach a power of.80 if employing *t*-tests.

## Results

### Completion

Thirty-six potential participants volunteered for the study. Four participants were excluded, one due to psychotic disorder, one due to ongoing substance abuse, and two participants because they failed to complete the pre-assessment. The remaining 32 participants were included in the study.

**FIGURE 1:**
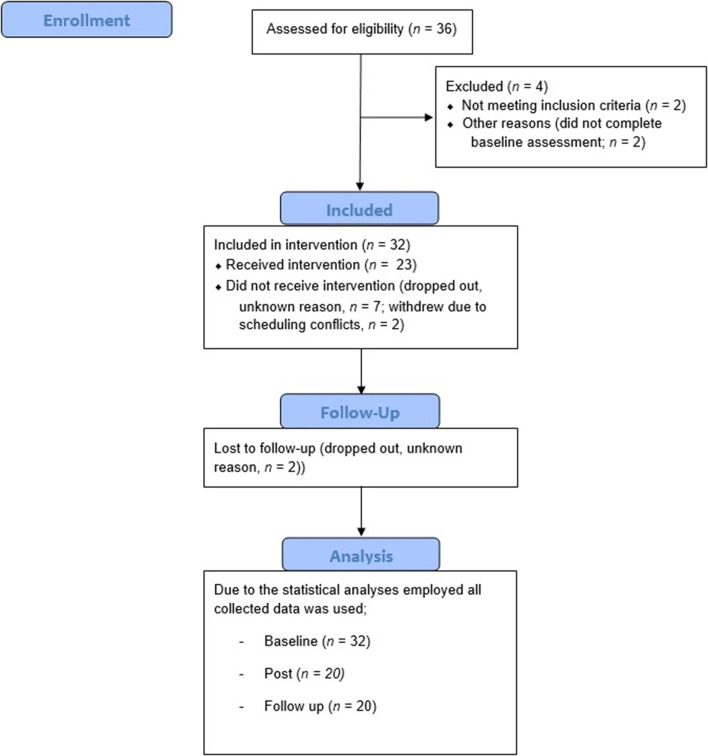
A diagram showing the enrollment of subjects, their inclusion in the treatment and the data included in the analyses.

Twenty-three participants completed the intervention, of which 21 completed the post-intervention and follow-up assessments. Of the 11 participants who dropped out, nine did so without giving notice or answering phone calls attempting to reestablish contact. Of these, one did so before the first treatment session, three participants attended one session prior to dropping out, one participant attended two sessions, two participants attended three sessions, and two participants completed the intervention but did not attend the post-treatment assessment. The two drop-out participants who gave notice both reported changes in their work schedules as the reason for drop-out after attending two and three sessions, respectively. All participants who completed the intervention attended a minimum of seven of the nine group sessions (see Figure 1).

### Missing Data and Outliers

The dataset had 16 missing single items from questionnaires (totaling 1% or less of total item responses per questionnaire). Little’s missing completely at random test showed that all missing items were randomly distributed. Missing items were therefore replaced using the expectation maximization algorithm in SPSS. One participants’ BRIEF-A-questionnaire from the pre-assessment was missing, assessment data from one participant was also missing for most measures of the post-assessment, and for another participant on all measures of the follow-up assessment. Complete questionnaires that were missing were not replaced.

In addition, 32 scores were identified as outliers (approximately 1% of total scores), and were replaced with ± MAD.

### Participant Characteristics

Table 2 shows baseline characteristics of the complete sample, in addition to the subgroups who completed the intervention (completers) and those who did not (non-completers). Non-completers were significantly younger and reported lower quality of life at baseline compared to completers. Regarding comorbidities there were no significant differences between completers and non-completers, but a non-significant trend toward non-completers reporting higher symptom severity.

**TABLE 2:** Descriptive characteristics of the sample and the subgroups of completers and non-completers at baseline.

	Total sample (*N* = 32)	Completers (*N* = 21)	Non-completers (*N* = 11)	*P*-values
Number of males (%)	18 (56.25%)	12 (57.14%)	6 (54.55%)	1.00
Age in years, mean (SD)	35.75 (11.87)	39.05 (11.93)	29.45 (9.25)	0.02^*^
Years of education, mean (SD)	13.88 (2.96)	14.33 (2.01)	13.00 (4.22)	0.34
Full-scale intelligence quotient, mean (SD)	118.25 (11.66)	120.14 (10.08)	114.64 (14.02)	0.27
Currently receiving medication, number (%)	16 (50.00%)	10 (47.62%)	6 (54.55%)	1.00
Currently receiving other therapy, number (%)	16 (50.00%)	9 (42.86%)	7 (63.64%)	0.46
Total ASRS score, mean (SD)	46.97 (10.07)	44.33 (8.91)	52.00 (10.63)	0.06
Total WURS score, mean (SD)	44.03 (19.44)	40.05 (16.55)	51.64 (22.97)	0.16
Comorbidities				
Mood disorders, ongoing. Number (%)	5 (15.62%)	2 (9.52%)	3 (27.27%)	0.37
Mood disorders, previous. Number (%)	28 (87.50%)	16 (76.19%)	12 (109.09%)	0.38
Anxiety disorders, ongoing. Number (%)	26 (81.25%)	18 (85.71%)	8 (72.73%)	0.39
Anxiety disorders, previous. Number (%)	16 (50.00%)	11 (52.38%)	5 (45.45%)	0.93
Alcohol or substance use disorders, previous. Number (%)	8 (25.00%)	6 (28.57%)	2 (18.18%)	0.07
Antisocial personality disorder, ongoing. Number (%)	4 (12.50%)	3 (14.29%)	1 (9.09%)	1.00
Other disorders, ongoing. Number (%)	8 (25.00%)	5 (23.81%)	3 (27.27%)	0.25
Self-reported executive difficulties				
BRIEF-A GEC, mean (SD)	147.03 (23.30)	142.57 (24.01)	155.55 (20.22)	0.12
CFQ, mean (SD)	61.41 (13.34)	58.33 (13.90)	67.27 (10.89)	0.06
Emotion regulation				
Total score – DERS, mean (SD)	97.91 (23.88)	95.19 (25.02)	103.09 (21.70)	0.36
Quality of life				
Total score – AAQoL, mean (SD)	49.89 (14.24)	54.19 (14.21)	41.69 (10.57)	0.01^**^
Executive functions				
Letter Number Sequencing, mean (SD)	19.38 (2.62)	19.57 (2.71)	19.00 (2.53)	0.56
Scaled scores, mean (SD)	10.41 (1.70)	10.62 (1.77)	10.00 (1.55)	0.36
Spatial span, mean (SD)	15.90 (2.72)	16.08 (2.82)	15.55 (2.62)	0.60
Scaled scores, mean (SD)	10.25 (2.33	10.57 (2.29)	9.64 (2.38)	0.32
CWIT. Condition 3 completion time, mean (SD)	55.58 (13.86)	54.26 (15.42)	58.09 (10.44)	0.41
Scaled scores, mean (SD)	9.69 (3.33)	10.19 (3.56)	8.73 (2.72)	0.19
CWIT. Condition 4 completion time, mean (SD)	62.47 (10.44)	61.19 (11.31)	64.91 (8.49)	0.31
Scaled scores, mean (SD)	9.47 (2.30)	9.86 (2.43)	8.73 (1.90)	0.08
CWIT. Total errors conditions 3 and 4, mean (SD) [*N* = 31/21/10]	2.21 (2.15)	2.02 (1.97)	2.60 (2.55)	0.54
TMT. Condition 4 Completion time, mean (SD)	70.98 (26.88)	69.97 (29.20)	72.91 (23.00)	0.76
Scaled scores, mean (SD)	10.38 (2.99)	10.67 (3.04)	9.82 (2.96)	0.56
TMT. Condition 4 Total errors, mean (SD)	0.75 (0.80)	0.86 (0.91)	0.55 (0.52)	0.23
Tower task. Total achievement, mean (SD)	19.36 (4.02)	19.12 (4.51)	19.82 (3.03)	0.61
Scaled scores, mean (SD)	11.69 (2.76)	11.62 (3.06)	11.82 (2.23)	0.53
Hotel task [*N* = 31/21/10]				
Total time deviation. Mean (SD)	342.26 (230.25)	330.96 (216.66)	366.00 (267.36)	0.72
Tasks attempted. Mean (SD)	4.71 (0.82)	4.71 (0.78)	4.70 (0.95)	
Total score, garage. Mean (SD)	6.51 (2.05)	6.65 (2.11)	6.21 (2.01)	0.58

*Full-scale intelligence quotient based on two subtests of the Wechsler Abbreviated scale of intelligence. ASRS, Adult ADHD Symptom Rating Scale; WURS, Wender-Utah Rating Scale for ADHD, other disorders include body dysmorphic disorder, bulimia nervosa, and premenstrual dysphoric disorder. BRIEF-A GEC, Global Executive Composite from the Behavior Rating Inventory of Executive Function; CFQ, Cognitive Failures Questionnaire; DERS, Dysregulation of Emotion Rating Scale; AAQoL, Adult ADHD Quality of Life inventory, Letter Number Sequencing from the Wechsler Adult Intelligence Scale – 4th edition, Spatial span from the Wechsler Memory Scale – 3rd edition. CWIT, Color-Word Interference Test; TMT, Trail Making Test, these and the Tower all from the Delis-Kaplan Executive Function System. Reported scaled scores have a normative mean of 10 and SDs of 3 for all reported measures (Delis et al., 2001; Wechsler, 2008b, a). *P*-values for continuous variables based on Welch’s two sample *t*-test, *p*-values for discrete variables based on Pearson’s Chi-squared test.*

^*^p < 0.05,

^**^p < 0.01.

### Treatment Effects – Completer Sample

An overview of changes in the included variables from pre- to post- and follow-up assessments among participants who completed GMT is shown in Table 3.

**TABLE 3:** Simplified outputs from Linear Mixed-Effects Regressions of performance-based measures examining change from baseline to post intervention and 6-month follow-up assessments.

	Session	β	SE	*t*	*p*		β	SE	*t*	*p*
**Primary measures**										
CWIT. Condition 3 completion time	Pre	55.55	2.14			Letter Number Sequencing, total score^§^	19.38	0.43		
	Post	−4.48	1.95	−2.30	0.022^*^		−0.82	0.43	−1.88	0.060
	Follow up	−5.60	1.98	−2.82	0.005^*^		−0.76	0.44	−1.72	0.086
CWIT. Condition 4 completion time	Pre	62.47	1.91			Spatial Span, total score^§^	15.93	0.50		
	Post	−8.64	2.00	−4.31	<0.001^*^		0.87	0.53	1.65	0.099
	Follow up	−9.33	2.04	−4.58	<0.001^*^		0.31	0.53	0.59	0.558
CWIT. Condition 3 and 4, total errors	Pre	2.37	0.38			Hotel task. Total time deviation	352.71	37.40		
	Post	−0.32	0.37	−0.89	0.375		−26.34	44.08	−0.60	0.550
	Follow up	−0.21	0.37	−0.57	0.570		−117.59	44.87	−2.62	0.009^*^
TMT. Condition 4. Completion time	Pre	72.03	4.81			Hotel task. Total tasks attempted^§^	4.71	0.11		
	Post	−7.78	4.54	−1.71	0.087		0.20	0.17	1.17	0.244
	Follow up	−11.37	4.63	−2.46	0.014^*^		0.29	0.17	1.71	0.087
TMT. Condition 4. Total errors	Pre	0.75	0.17			Hotel task. Total score, garage^§^	6.10	0.44		
	Post	−0.16	0.24	−0.67	0.503		0.84	0.48	1.77	0.076
	Follow up	0.06	0.24	0.22	0.822		0.34	0.48	0.70	0.484
Tower task. Total achievement^§^	Pre	19.38	0.68							
	Post	0.30	0.70	0.42	0.672					
	Follow up	2.12	0.71	2.99	0.003^*^					

*Letter Number Sequencing from the Wechsler Adult Intelligence Scale – 4th edition, Spatial span from the Wechsler Memory Scale – 3rd edition. CWIT, Color-Word Interference Test; TMT, Trail Making Test, these and the Tower all from the Delis-Kaplan Executive Function System. *P*-values estimated using Satterthwaite’s method.*

^*^p 0.05 after application of control for false discovery rates.

*^§^Hypothesized increase in scores from pre-assessment, remaining measures are hypothesized to decrease.*

#### Primary Outcome Measures – Performance-Based Tests of Executive Functions

Analyses of scores on the performance-based measures of executive functions and problem solving showed improved efficiency in inhibitory control from baseline to post-assessment, as evidenced by a significant reduction in completion times on the third and fourth conditions of the CWIT (*M*s = 55.55 and 62.47 at baseline, *M*dif = −4.48 and −8.64 for the third condition and fourth condition, respectively). These changes were maintained at follow-up 6 months later (*M*dif = −5.60 and −9.33, respectively). The participants also increased their achievement scores on the Tower test at the follow-up assessment relative to baseline (*M* = 19.38 at baseline, *M*dif = 2.12 at follow-up) and improved their performance on the fourth condition of the TMT (*M* = 72.03 at baseline, *M*dif = −11.37 at follow-up), thus showing further improvements in inhibition in addition to attentional control and cognitive flexibility. No significant changes were detected on the other performance-based measures of executive function. However, on the Hotel task, an improvement in general problem solving was shown in a reduction in deviation from the ideal time from pre- to follow-up assessment (*M* = 352.71 at baseline, *M*dif = −117.59 at follow-up).

The exploratory analyses investigating the potential effects of age and medication status did not show any significant effects of these covariates (all *p*s ≥ 0.09 without correction for multiple comparisons).

#### Secondary Outcome Measures of Self-Reported Symptoms, Quality of Life, and Everyday Functioning

As secondary effects, the participants reported a significant reduction of cognitive functioning difficulties in their everyday lives, as measured by the CFQ (*M* = 61.41 at baseline, *M*dif = −6.75 and −7.61 at post and follow-up, respectively). The participants further reported a significant reduction of ADHD symptoms on the ASRS at both time points. They also reported increased quality of life following GMT as measured by the Life productivity subscale of the AAQoL (*M* = 42.32 at baseline, *M*dif = 11.89 and 12.52 at post and follow-up, respectively). This subscale assesses functioning in school/work and everyday task accomplishment. The remaining subscales of the AAQoL showed no significant changes, but there was a significant change to the total score (*M* = 49.36 at baseline, *M*dif = 6.95 and 8.24 at post and follow-up, respectively). Please see Table 4 for further information.

**TABLE 4:** Simplified outputs from linear mixed-effects regressions of self-report measures examining change in ADHD-symptoms, everyday cognitive functioning and quality of life from baseline to post intervention and 6-month follow-up assessments.

	Session	β	SE	*t*	*p*
**Secondary measures**					
ASRS total score	Pre	46.97	1.85		
	Post	−3.70	1.52	−2.43	0.015^*^
	Follow up	−5.28	1.52	−3.47	<0.001^*^
CFQ total score	Pre	61.41	2.43		
	Post	−6.75	2.09	−3.23	0.001^*^
	Follow up	−7.61	2.09	−3.65	<0.001^*^
AAQoL Life Outlook	Pre	53.13	2.70		
	Post	5.38	2.67	2.02	0.044
	Follow up	5.02	2.67	1.88	0.060
AAQoL Life Productivity	Pre	42.32	3.01		
	Post	11.89	3.79	3.14	0.002^*^
	Follow up	12.52	3.79	3.31	<0.001^*^
AAQoL Psychological Health	Pre	54.30	3.65		
	Post	0.43	4.17	0.10	0.917
	Follow up	4.87	4.17	1.17	0.243
AAQoL Relationships	Pre	56.72	3.82		
	Post	2.73	4.09	0.67	0.504
	Follow up	3,97	4.09	0.97	0.331
AAQoL total score	Pre	49.36	2.64		
	Post	6.95	3.03	2.30	0.022^*^
	Follow up	8.24	3.03	2.72	0.007^*^

*ASRS, Adult ADHD Symptom Rating Scale; CFQ, Cognitive Failures Questionnaire; AAQoL, Adult ADHD Quality of Life inventory. *P*-values estimated using Satterthwaite’s method.*

^*^p < 0.05 after application of control for false discovery rates.

In addition to these self-reported changes, the participants also reported significant improvements on measures of everyday executive functioning and aspects of emotion regulation (see Supplementary Table 1).

## Discussion

The main aim of the current study was to conduct an exploratory pilot testing of a neuropsychological model for examining the effects of GMT on inhibition, specifically, in a sample of adults with ADHD. The effects of GMT were studied immediately after completing the treatment and at a 6-month follow-up assessment. Due to the emphasis in GMT on strategies supporting the executive function of inhibition (Levine et al., 2000, 2011), we expected that measures of this, and not other aspects of executive functioning such as working memory, flexible control of processing speed or general problem solving, would show significant improvement. Indeed, we found support for this hypothesis in that the adults with ADHD demonstrated improved inhibitory control on selected neuropsychological measures of inhibition after completing GMT both at the post- and 6-month follow-up assessments, but not on tests of working memory. Interestingly, at follow-up after 6 months, the results also showed improved problem-solving skills on the Hotel task and improvements on a measure of flexible control of processing speed. In addition to the improvements in inhibitory control following GMT, we found secondary positive effects after GMT in that the adults with ADHD reported improvements in everyday functioning. This was shown through self-reported improvements in ADHD-symptoms, everyday cognitive functioning and quality of life. Participants also reported improvements in aspects of executive functioning and emotion regulation, as can be seen in the Supplementary Materials.

In GMT, participants work specifically on improving strategies supporting goal-directed behavior by practicing intermittent stopping of ongoing behavior to monitor whether this behavior is in line with current goals (Levine et al., 2011; see also Cooper, 2010). Thus, GMT emphasizes inhibitory control training (i.e., “STOP!-and-think”). The results in the current study supported our hypothesis that this specific function would improve following GMT compared to other neuropsychological measures of executive functions. Improvements in inhibition were evident both immediately following the intervention and 6 months later on the CWIT measure of interference control, a subtest requiring voluntary control over which stimuli are attended. Furthermore, after 6 months, improvements were also observed on the Tower test. This is a problem-solving test specifically requiring inhibitory control to be able to follow the rules and complete the tower as instructed (see Miyake et al., 2000; Young et al., 2007). The importance of strengthening inhibition is reflected in both theories of ADHD, suggesting that impaired inhibition is a predominant cause of the negative impact on everyday functioning associated with the disorder (e.g., Barkley, 1997), as well as in findings showing its importance for general functioning. Inhibition is central for the ability to pursue goal-directed behavior (Cooper, 2010), which is required in academic work and in occupational work settings (e.g., Halleland et al., 2015). It is also shown to be important for emotion regulation (see Shaw et al., 2014). Furthermore, there is research pointing to inhibitory control as an important aspect of psychological resilience (see Kalisch et al., 2015), which reduces the risk of developing adverse, psychological reactions despite exposure to stressful and potentially traumatic events.

Due to the importance of inhibitory control in regulating behavior, we expected that GMT would lead to improvements in the ability to handle the challenges of everyday life. Therefore, we expected positive secondary effects of GMT on self-reported everyday functioning. The results of the current study are in accordance with this expectation as the adults with ADHD reported improvements on self-reports of cognitive and executive functioning. In particular, participants reported improvements on aspects of such functioning related to increased productivity and aspects of controlled emotion regulation. Further, positive effects of GMT were reported as a perceived reduction in the severity of ADHD symptoms after 6 months, and increased productivity with regards to school/work and everyday task achievement (AAQoL). Reports on the DERS questionnaire 6 months after completing GMT also showed that the participants experienced an enhanced ability to regulate emotional responses and were better able to use active strategies for helping them when they experienced negative emotions.

In line with our expectations, the adults with ADHD did not show changes in working memory after completing GMT. Interestingly, however, we found that after 6 months the adults with ADHD improved their general problem-solving skills as well as their flexible control of processing speed. Although we did not expect this, we believe that this change supports the interpretation of GMT leading to functional improvements. This is in contrast to the prior pilot study of an adapted version of GMT in adults with ADHD in which no change following the intervention was found on an everyday problem-solving task (In de Braek et al., 2017). The Hotel task is meant to be an analog of executive functioning in complex everyday situations (Shallice and Burgess, 1991; Manly et al., 2002), and requires that the participant devices a plan for performing the task whilst simultaneously monitoring his or her behavior and the time remaining. There are findings suggesting that the improvements on the Hotel task after completing GMT in the current study may be seen, at least in part, as a result of internalization of the exercises focusing on intermittent stopping (e.g., “STOP!-and-think”) by the participants. The periodic suspension of ongoing behavior to evaluate one’s overarching goal seems to increase goal achievement (Manly et al., 2002). In other patient samples, GMT has also been shown to improve performance on the Hotel task and similar analogs of real-life task performance (e.g., Levine et al., 2000, 2007; Miotto et al., 2009; Novakovic-Agopian et al., 2011; Stubberud et al., 2013; Tornås et al., 2016). Furthermore, in line with the findings from the current study, the majority of these studies have also shown improvements in self- or informant-reported evaluations of everyday functioning, which would be expected if participants had indeed internalized an efficient problem-solving strategy. Interestingly, in a study that showed no effect of GMT on an everyday problem-solving task, the participants did not report changes following GMT on measures of everyday cognitive functioning (Levine et al., 2011).

As the current study was a self-control case design, we cannot rule out that practice effects may have contributed to improved scores from pre- to post- and follow-up assessments. Important to note in this regard, is the fact that we hypothesized that inhibitory control would be improved, and found that neuropsychological test measures of this function, and not of working memory, improved after completing GMT. We believe that this supports the assumption that our primary results of enhanced inhibitory control are not due to pure practice effects. This interpretation is supported by results from available studies of practice effects. For instance, Calamia et al. (2012) found similar retest effect sizes for measures of working memory, processing speed and more general executive functions in their meta-analysis. Several meta-analyses have also shown that practice effects are most pronounced between the first and second administration of a test, with smaller increases for subsequent administrations (Scharfen et al., 2018a,b). This is important since we found strong effects 6 months after, and not just immediately after, completing GMT. The meta-analysis of Calamia et al. (2012) also showed that practice effects were substantially less pronounced for clinical samples compared to healthy samples.

Although the findings in the current study need to be interpreted with caution, the results strongly support the notion that the participants with ADHD adopted the learned strategies and applied them in their everyday life 6 months after completing GMT. Furthermore, in line with the primary aim of the study, our findings support the use of neuropsychological outcome variables as effect measures of GMT in ADHD samples. Specifically, GMT in ADHD seems to address inhibitory control in particular more than executive functioning in general. Interestingly, newer revisions of the understanding of executive functions lean toward inhibitory control being the unitary component of executive functioning (Cooper, 2010; Miyake and Friedman, 2012). This may imply that the neuropsychological model for assessing effect of GMT in the current study can be applicable also for studies testing the effect of GMT in other clinical samples than ADHD. A critical point of treatment studies in ADHD is the question of whether the effects are generalized to the patients’ everyday life. Cognitive remediation approaches have often been criticized for failing on this point in ADHD samples, examples include working memory training and neurofeedback (see Melby-Lervåg and Hulme, 2013; Cortese et al., 2016). In the current study, we found that the adults with ADHD also experienced improvement in their everyday life, lasting at least 6 months after completion of GMT. Future studies are, however, needed to test if these findings are replicated with a case-control design. Applying a case-control design would allow for control for spontaneous changes in how patients experience their life or changes associated with non-specific effects (e.g., professional attention, group dynamics; McCambridge et al., 2012). Since the current study aimed to explore test-effects of GMT by assessing the patients with ADHD with a neuropsychological test battery at three time points, resources for testing were prioritized above recruiting a bigger sample of patients. Due to potential participants being excluded, withdrawing due to scheduling conflicts and drop-out, the final number of participants included was not in line with the original plans for the project and the power analysis conducted during this planning. As such, lower power in the statistical analyses may have contributed to negative results on effect measures that with a larger sample would appear as a positive effect of GMT. Future studies may therefore identify effects on measures which did not reach significance in the current study. Of note, we did control for multiple testing in our statistical analyses, and the effects of GMT on inhibitory control measures were still strong enough to reach significance. Also, of relevance, the baseline data showed that the drop-out group was younger than the patients completing the GMT and the post-assessments, and also a tendency for the drop-out group to report higher symptom severity. In treatment studies of ADHD, there is often a problem with patients dropping out, which can result in a biased sample of patients completing the treatment. This can be handled with intention-to-treat analyses, however, this was not possible in the current study due to the self-control case design.

The current study indicates significant effects of GMT on inhibition among adults with ADHD. Furthermore, the current results provide support for the notion that GMT may also affect broad and important domains of functioning such as everyday cognition and productivity as well as emotion regulation. These results encourage further studies that include control conditions to examine GMT as an intervention for adults with ADHD, as replication would indicate that GMT represents an efficient and cost-effective treatment alternative.

## Data Availability Statement

The datasets presented in this article are not readily available because the ethical approval states that anonymized data can only be made available for registered collaborators. Requests to access the datasets should be directed to DJ, Daniel.A.Jensen@uib.no.

## Ethics Statement

The studies involving human participants were reviewed and approved by Regional Committee for Medical and Health Research Ethics, West Norway. The patients/participants provided their written informed consent to participate in this study.

## Author Contributions

LS was the project leader. DJ, LS, AL, AH, JS, and JH involved in conception and design, and approved the final version for publication. DJ collected the data. DJ and LS involved in analyses, interpretation, and writing of the manuscript. AH, AL, JS, and JH critically reviewed the manuscript.

## Conflict of Interest

JH has received speaker honoraria from Lilly, Shire, HB Pharma, Medice, Takeda, and Biocodex. The remaining authors declare that the research was conducted in the absence of any commercial or financial relationships that could be construed as a potential conflict of interest.

## Publisher’s Note

All claims expressed in this article are solely those of the authors and do not necessarily represent those of their affiliated organizations, or those of the publisher, the editors and the reviewers. Any product that may be evaluated in this article, or claim that may be made by its manufacturer, is not guaranteed or endorsed by the publisher.
