# Supplementary material for: An Exploratory Investigation of Goal Management Training in Adults With ADHD: Improvements in Inhibition and Everyday Functioning
Source: Front Psychol. 2021 Sep 9;12:659480. doi: 10.3389/fpsyg.2021.659480 (PMC8458564; doi:10.3389/fpsyg.2021.659480)
Supplement: Supplementary file 1 [file Table_1.DOCX]

Supplementary Material

**Supplementary Table 1:** Simplified outputs from linear mixed-effects regressions of self-report measures of self-regulation examining change from baseline to post intervention and six-month follow-up assessments.

|  | Session | β | | SE | t | p |  |  |  | β | SE | t | p | |  |
| --- | --- | --- | --- | --- | --- | --- | --- | --- | --- | --- | --- | --- | --- | --- | --- |
| **Measures of self-regulation** |  |  |  | |  |  |  |  |  |  |  |  |  | |  |
| BRIEF-A Inhibit | Pre | 16.22 | 0.62 | |  |  |  |  | DERS Difficulty engaging in Goal-directed behavior (GOALS) | 18.13 | 0.87 |  | |  |  |
|  | Post | -1.36 | 0.60 | | -2.26 | 0.024 | * |  |  | -0.69 | 0.70 | -0.97 | | 0.330 |  |
|  | Follow up | -1.60 | 0.60 | | -2.66 | 0.008 | * |  |  | -1.88 | 0.70. | -2.68 | | 0.008 | * |
| BRIEF-A Shift | Pre | 11.95 | 0.44 | |  |  |  |  | DERS Impulse control difficulties (IMPULSE) | 15.44 | 0.83 |  |  | |  |
|  | Post | -1.16 | 0.54 | | -2.16 | 0.031 |  |  |  | -2.16 | 0.86 | -2.51 | 0.012 | | * |
|  | Follow up | -0.63 | 0.54 | | -1.18 | 0.237 |  |  |  | -2.51 | 0.86 | -2.91 | 0.004 | | * |
| BRIEF-A Working Memory | Pre | 18.91 | 0.55 | |  |  |  |  | DERS Limited access to emotion regulation strategies (STRATEGIES) | 19.49 | 1.12 |  |  | |  |
|  | Post | -1.62 | 0.66 | | -2.46 | 0.014 | * |  |  | -2.05 | 0.96 | -2.14 | 0.032 | |  |
|  | Follow up | -2.57 | 0.66 | | -3.91 | <0.001 | * |  |  | -3.35 | 0.96 | -3.50 | <0.001 | | * |
| BRIEF-A Behavior Regulation Index | Pre | 58.16 | 2.04 | |  |  |  |  | DERS Total score | 97.91 | 4.25 |  |  | |  |
|  | Post | -4.40 | 1.92 | | -2.29 | 0.022 | * |  |  | -3.56 | 3.86 | -0.92 | 0.356 | |  |
|  | Follow up | -4.31 | 1.92 | | -2.25 | 0.025 | * |  |  | -10.56 | 3.86 | -2.74 | 0.006 | | * |
| BRIEF-A Metacognition Index | Pre | 89.91 | 2.64 | |  |  |  |  |  |  |  |  |  |  |  |
|  | Post | -8.64 | 2.75 | | -3.15 | 0.002 | * |  |  |  |  |  |  |  |  |
|  | Follow up | -11.12 | 2.75 | | -4.05 | <0.001 | * |  |  |  |  |  |  |  |  |
| BRIEF-A Global Executive Composite | Pre | 147.03 | 4.33 | |  |  |  |  |  |  |  |  |  |  |  |
|  | Post | -11.67 | 4.44 | | -2.63 | 0.009 | * |  |  |  |  |  |  |  |  |
|  | Follow up | -14.08 | 4.44 | | -3.17 | 0.002 | * |  |  |  |  |  |  |  |  |
| ^Note: BRIEF-A = Behavior Rating Inventory of Executive Function, DERS = Dysregulation of Emotion Rating Scale. P-values estimated using Satterthwaite's method. * =^ *^p^* ^< 0.05 after application of control for false discovery rates.^ | | | | | | | | | | | | | | | |
|  |  |  |  |  |  |  |  |  |  |  |  |  |  |  |  |
|  |  |  |  |  |  |  |  |  |  |  |  |  |  |  |  |
